# Three cases of alloimmune mediated pancytopenia in calves resembling bovine neonatal pancytopenia

**DOI:** 10.1186/s12917-021-03117-z

**Published:** 2022-01-03

**Authors:** L. Chantillon, B. Devriendt, B. De Jonge, J. Oostvogels, J. Coppens, M. L. Pas, J. Bokma, B. Pardon

**Affiliations:** 1 Department of Large Animal Internal Medicine, Faculty of Veterinary Medicine, Ghent University, Salisburylaan 133, 9820 Merelbeke, Belgium; 2 Laboratory for Immunology, Department of Virology, Parasitology and Immunology, Faculty of Veterinary Medicine, Ghent University, Salisburylaan 133, 9820 Merelbeke, Belgium; 3 Department of Pathology, Bacteriology and Poultry Diseases, Faculty of Veterinary Medicine, Ghent University, Salisburylaan 133, 9820 Merelbeke, Belgium; 4 Veterinary Practice Venhei, Geelsebaan 95-97, 2460 Kasterlee, Belgium

**Keywords:** Vaccination- bleeding- immunopathology- thrombocytopenia- alloantibodies- Belgian blue calves

## Abstract

**Background:**

Between 2007 and 2011 several thousands of calves died from bovine neonatal pancytopenia (BNP), a bleeding syndrome triggered by vaccine induced alloantibodies from the dams. Following withdrawal of the involved bovine viral diarrhoea virus (BVDv) vaccine, the incidence of this condition rapidly decreased, with no reported cases in the last 5 years. Here, we report a recent immune-mediated pancytopenia in three calves from two different suckler herds, clinically indistinguishable from BNP.

**Case presentation:**

Three Belgian Blue suckler calves from two different farms, aged around two weeks, showed multiple bleedings disseminated on the skin and petechiae and ecchymoses on the mucosae. Blood examination confirmed anaemia, leukopenia and thrombocytopenia. BVDv infection was excluded. Despite blood transfusion and cortisone therapy, all three animals died. Necropsy and histology confirmed bone marrow depletion. Binding of IgG from the dams on leukocytes of the calves was demonstrated by flow cytometry. Two calves, originating from the same farm, received colostrum from the same dam. None of the calves were given colostrum replacers or colostrum supplements. No link with the BNP causing BVDv vaccine could be evidenced. However, dams had been vaccinated against bovine herpesvirus 1, parainfluenza-3 virus, bovine respiratory syncytial virus and bluetongue virus serotype 8.

**Conclusions:**

Alloimmune mediated pancytopenia was evidenced in three animals, clinically and pathologically indistinguishable from BNP. Whether this disease is again vaccine mediated remains to be determined.

## Background

Between 2007 and 2011, in Europe, and to a lesser extent in other continents, thousands of calves died due to the consequences of bovine neonatal pancytopenia (BNP) [1–11]. BNP is a bleeding disorder, typically developing between 10 and 21 days after birth, with severe internal and external bleeding (skin bleeding and petechia on mucosae) due to thrombocytopenia [3, 12]. The clinical condition rapidly deteriorates and animals die either due to haemorrhagic or septic shock [12]. The characteristic haematological finding is a pancytopenia starting with thrombocytopenia, followed by neutropenia, lymphopenia and non-regenerative anaemia [3, 9, 12–16]. At post-mortem examination, consistently a complete depletion of the bone marrow from all cell populations is found [17–19]. BNP’s clinical manifestation can range from hyperacute with high mortality to subclinical [3, 20]. Variability in severity could be explained by differences in the quantity of ingested alloantibodies, genetics of dam, sire and calf and the lag time between birth and ingestion of colostrum [18, 21]. The causal role of a bovine viral diarrhea virus vaccine (PregSure® BVD, Pfizer Animal Health, Berlin, Germany) in BNP was evidenced [2, 4, 13, 22–26]. The adjuvanted vaccine induced the production of alloantibodies in some vaccinated cattle depending on their genotype of bovine major histocompatibility complex class I [2, 4, 21–25, 27–29]. Through pooling of colostrum or by the use of colostrum derivatives (colostrum supplements and replacers) multiple calves could be affected with the colostrum of one dam. The responsible vaccine was withdrawn from the market in 2010, and gradually the number of BNP cases declined to almost zero over a 5-year period or in some regions even longer [2, 30, 31].

Also before the emergence of BNP, bleeding disorders in calves occurred, albeit at low frequency. Multiple etiologies of these bleeding disorders have been described, like bacterial sepsis or viral (e.g. BVDv) infections, intoxications or hereditary diseases [12]. Reporting continues today, unfortunately often without a final diagnosis. To what extent immunological tests are nowadays routinely used in these sporadic cases of bleeding disorders is unknown to the authors. Here, we report three cases of alloantibody mediated pancytopenia in suckler calves, resembling BNP.

## Case presentation

### Case history

In August 2020, two female Belgian Blue calves were presented at the clinic of internal medicine in a critically ill state with complaints of skin bleeding. Case 1 and 2 were twelve and eight days old respectively and housed in the same stable with their mothers. The owner reported that the oldest calf started bleeding a few days earlier on skin of the ears, with trauma being the suspected cause. No fever was present. Treatment with gentamicin and penicillin by the herd veterinarian gave no clinical improvement. During the evening before admission, the clinical condition of case 1 deteriorated severely with development of dyspnoea. The owner noticed blood in the faeces, on the skin and petechiae and ecchymoses on oral and eye mucosae. Another calf started to show similar signs with skin bleeding, dyspnoea and also no fever. Both cases received one litre of a Hartmann-glucose 5% infusion from the veterinarian before referral to the clinic. Although they were born four days apart, both had received colostrum from the mother of case 1. The case dam was born in 2013 and had received different vaccinations throughout the years. As a calf, she was intranasally vaccinated against respiratory syncytial virus (bRSV) and parainfluenza virus type 3 (PI-3) (Rispoval intranasal®, Zoetis B.V., Capellen aan den Ijssel, The Netherlands) at about two weeks of age and then another vaccine against bRSV, Pi-3 and BVDv (Rispoval 3®, Zoetis Belgium SA, Louvain-la-Neuve, Belgium) at 3 and 4 months of age. Subsequently, she was vaccinated for bovine herpesvirus 1 with Bovilis IBR Marker Live® (Intervet Nederland B.V., Boxmeer, The Netherlands) annually until 2018 and against bluetongue virus serotype 8 (BTV-8) with Bluevac BTV® vaccine (CZ Veterinaria S.A., Porriño, Spain) annually until 2019. In 2020, the bluetongue vaccine was changed and the dam received Bovilis Blue-8® (Intervet International B.V., Boxmeer, The Netherlands). Also, since 2017, she was vaccinated annually against enterotoxigenic *Escherichia coli*, rotavirus and coronavirus with Rotavec® (Intervet International B.V., Boxmeer, Nederland). Before 2017, she was vaccinated annually for the same pathogens with Coroniffa® (Boehringer Ingelheim animal health France, Lyon, France). No problems were noticed during previous parturitions and also the mother of case 2, born in 2017, delivered a healthy calf the year before. The two affected calves descent from different bulls.

The third case, a fourteen-day old male calf admitted in August 2021, had a similar clinical appearance. For five days he showed bloody faeces, bleeding on the skin of the ears and body, nose bleeding and petechiae on mucosae. The calf was still suckling and alert and no treatment was established before admission. He received colostrum from a dam other than his mother. The herd, which has no connection with the herd of case 1 and 2, had already been confronted with two bleeding calves in the past year, all of whom received colostrum from the same dam as case 3. Both calves died from the bleeding disorder and were not admitted to the clinic. One of these calves was descended from the mother of case 3 although her first calf did not show any abnormalities or disease. Case 3 did not have the same sire as case 1 or 2 and sires of the two other affected calves in this herd could not be determined. The colostrum donating dam was born in March 2017 and received an intranasal vaccine against bRSV and PI-3 (Rispoval intranasal®, Zoetis B.V., Capellen aan den Ijssel, The Netherlands) and an intramuscular vaccine against *Mannheimia haemolytica* (Pastobov®, Boehringer Ingelheim Animal Health Belgium SA, Brussels, Belgium) at one month of age. A booster with Pastobov® was injected a month later. Five and six months after birth, she had been vaccinated against bRSV, Pi-3 and BVDv (Rispoval 3®, Zoetis Belgium SA, Louvain-la-Neuve, Belgium). Subsequently an annual vaccination against BVDv (Bovela®, Boehringer Ingelheim Vetmedica GmbH, Ingelheim/Rhein, Germany) and against BTV-8 (Bluevac BTV®, CZ Veterinaria S.A., Porriño, Spain) was conducted. Since 2020 Bluevac BTV® was replaced by Bovilis Blue-8® (Intervet International B.V., Boxmeer, The Netherlands).

### Clinical examination

On arrival at the clinic, a clinical examination of all three cases was carried out by the same clinician. Results can be consulted in Table 1. All three calves showed disseminated skin bleedings and multiple petechiae and ecchymoses on the mucosae of the mouth and eyes.

**Table 1:** Overview of the clinical examination of case 1, case 2 and case 3

	Case 1	Case 2	Case 3
Body temperature (°C)	40.1	39.6	40.8
Pulse (bpm)	100	80	140
Respiration rate (breaths/min)	60	44	68
State	Lethargic	Alert	Alert
Mucosae	Pale, moist	Pale pink	Pale
Capillary refill time (s)	>2	>2	<2
Skin turgor	Reduced	Reduced	Reduced
Vena jugularis	Slow filling	Slow filling	Normal filling
Auscultation heart	Normal	Normal	Normal
Auscultation lung	enhanced respiratory sounds	enhanced respiratory sounds	enhanced respiratory sounds
Auscultation abdomen	Sloshing sounds	Sloshing sounds	Normal
Faeces	Aqueous, melena, fresh blood	Aqueous, melena, fresh blood	Aqueous, melena, fresh blood
Bodyweight	57	60	56

Ultrasonography was performed with a linear 7.5 MHz probe (Easote MyLab™30 Gold unit, the Netherlands) and 75% isopropanol solution (propanol-2, Chem Lab NV, Zedelgem, Belgium) as transducer agent between probe and skin. Case 1 and 2 showed some comet tails (B-lines) dispersed over the lung field and enlarged kidneys with a hypoechogenic medulla. Small intestines were immotile, but not dilatated. No other abnormalities were found. Case 3 showed B-lines at the cranial lung lobes. Small intestines were motile and the kidneys were normal. Only a mild omphaloarteritis was diagnosed.

### Blood examination

After clinical examination, a blood-gas analysis and a full blood count of all three cases was conducted. All blood samples were taken from the jugular vein with a vacutainer system (Venoject®, Terumo, Leuven, Belgium). Blood-gas analysis was performed on heparin blood tubes and analysed with RAPIDPoint® 405 (Siemens Healthcare, Beersel, Belgium). Haematology was executed on standard ethylenediaminetetraacetic acid blood tubes with the IDEXX ProCyte Dx Hematology Analyzer® (IDEXX Europe B.V., Hoofddorp, The Netherlands) and biochemistry on serum with the IDEXX Catalyst One Chemistry Analyser® (IDEXX Europe B.V., Hoofddorp, The Netherlands). The determination of the coagulation times in case 3 was done on a sodium citrate tube by an external laboratory. In Table 2 an overview of available blood parameters is given. Total bilirubin, total protein and gamma-glutamyltransferase were not measured in case 1 as the calf died shortly after arrival and only a limited amount of serum was collected.

**Table 2:** Blood-gas analysis, full blood count and clinical chemistry of case 1, case 2 and case 3

Parameter	Case 1	Case 2	Case 3	Reference interval
**Blood-gas analysis**^a^				
pH	7.14	7.37	7.45	7.350–7.450
pCO_2_	41.9	34.6	41.3	35–45 mmHg
HCO_3_	14.0	19.7	28.1	25–30 mmol/L
Hematocrit	11	15	12	25–35%
Base excess	−13.4	−5.1	3.8	−5 - +5 meq/L
Glucose	<20	76	79	60–80 mg/dL
Lactate	24.79	0.54	2.38	<2 mmol/L
Na^+^	142.5	133.9	135	132–152 mmol/L
K^+^	4.5	4.2	3.99	3.5–4 mmol/L
Ca^2+^	1.14	1.2	1.16	1.0 mmol/L
Cl^−^	99	103	99	100 mmol/L
**Complete blood count**^b^				
Red blood cell count	2.00 x 10^12^	3.32 x 10^12^	2.82 x 10^12^	4.47–9.35 x 10^12^/L
Hematocrit	7.7	11.8	10.7	22.5–39.9%
Hemoglobin	2.9	4.9	3.7	7.4–12.8 g/dL
Mean corpuscular volume	38.5	35.5	37.9	40.4–56.4 fL
Mean corpuscular hemoglobin	14.5	14.8	13.1	11.5–18.5 pg
Mean corpuscular hemoglobin concentration	37.7	41.5	34.6	30.2–33.5 g/dL
Red cell distribution width	30.2	31.1	36.1	20.0–35.9
%Reticulocytes	0.0%	0.0%	0.0	
Reticulocytes	0.2	0.0	0.0	0.0–3.9 K/μL
White blood cell count	0.36 x 10^9^	0.17 x 10^9^	0.97 x 10^9^	2.71–17.76 x 10^9^/L
%Neutrophils	50.0%	47.0%	3.1%	
%Lymphocytes	44.4%	47.1%	90.7%	
%Monocytes	2.8%	0.0%	6.2%	
%Eosinophils	2.8%	5.9%	0.0%	
%Basophils	0.0%	0.0%	0.0%	
Neutrophils	0.18 x 10^9^	0.08 x 10^9^	0.03 x 10^9^	0.68–6.94 x 10^9^/L
Lymphocytes	0.16 x 10^9^	0.08 x 10^9^	0.88 x 10^9^	1.20–10.62 x 10^9^/L
Monocytes	0.01 x 10^9^	0.00 x 10^9^	0.06 x 10^9^	0.02–2.17 x 10^9^/L
Eosinophils	0.01 x 10^9^	0.01 x 10^9^	0.00 x 10^9^	0.01–1.23 x 10^9^/L
Basophils	0.00 x 10^9^	0.00 x 10^9^	0.00 x 10^9^	0.00–0.04 x 10^9^/L
Platelet count	0	0	0	147–663 K/μL
Mean platelet count	9.4	8.2	10.3	5.9–8.2 fL
Procalcitonin	0.00%	0.00	0.00	0.12–0.42%
**Biochemistry**^b^				
Creatinine	230	188	210	0–172 μmol/L
Urea	6.8	4.8	5.5	2.5–6.1 mmol/L
Total bilirubin	Not available	<2	<2	0–12 μmol/l
GGT	Not available	23	56	0–80 IU/L
Total protein	Not available	33	44	58–80 G/L
**Coagulation indicators**^c^				
Prothrombin time	Not available	Not available	49.1	24–36 s
Activated partial thromboplastin time	Not available	Not available	33.3	24–35 s
Fibrinogen	Not available	Not available	489	100–460 mg/dL
D-dimer	Not available	Not available	190	<500 ng/mL

^a^Reference values obtained from Dillane et al. (2018) [32]

^b^Reference values obtained by the manufacturer

^c^Reference values obtained by the extern laboratory

Case 1 presented with severe metabolic acidosis, hypoglycaemia and hyperlactatemia. All three calves were severely leukopenic, thrombocytopenic and anaemic, confirming pancytopenia. Creatinine was increased, likely due to dehydration, and total protein was too low in case 2 and 3.

Sepsis couldn’t be excluded, so blood was aseptically sampled for haemoculture. Two different aerobic BD BACTEC™ blood culture media (Peds Plus™ and Plus Aerobic medium™, BD, Erembodegem, Belgium) were used in all three cases and in addition an anaerobic and a mycobacterium, yeast and fungi BD BACTEC™ blood culture (Lytic Anaerobic medium™ and Myco/F Lytic Culture Vials™, BD, Erembodegem, Belgium) was used in case 3. The blood cultures were subsequently incubated at 35 °C in an automated system for the detection of microbial growth (BACTEC™ FX). After two days, two BD BACTEC™ Peds Plus™ and one BD BACTEC™ Plus Aerobic medium™ marked positive and were send to an external laboratory for identification and susceptibility testing by the disk diffusion method. Final identification was done by matrix assisted laser desorption/ionisation time-of-flight mass spectrometry (MALDI-TOF MS). *Globicatella sanguinis* was isolated from case 1, *Staphylococcus chromogenes* from case 2 and *Staphylococcus xylosus *and* Sphingomonas paucimobilis* from case 3. Except for *Sphingomonas paucimobilis*, all bacteria were multidrug resistant (MDR), defined as resistance to agents from at least three different antimicrobial classes [33] (data not shown). The Lytic Anaerobic medium™ and Myco/F Lytic Culture Vials™ blood cultures showed no growth and remained negative.

All animals were ear notch tested for BVDv antigen by an indirect antigen-capture enzyme-linked immunosorbent assays (ELISA) (BVDv Ag/Serum Plus Test, IDEXX Laboratories, Inc., USA), as part of the Belgian BVDv eradication program. BVDv was not detected in any of the tested samples.

### Antibody binding assay

**Fig. 1:**
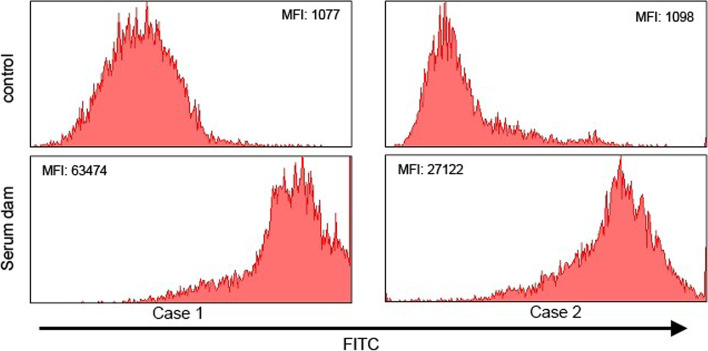
Leukocytes from two calves (case 1 and 2) were stained with serum of their dams or PBS (control) and an FITC-conjugated anti-bovine IgG. The mean fluorescence intensities (MFI) of the leukocytes after incubation with the sera from both dams of case 1 (left) and case 2 (right) were compared with these following incubation with PBS. The MFI values, given in the histograms, of the sera from the cases were higher than those from both controls

**Fig. 2:**
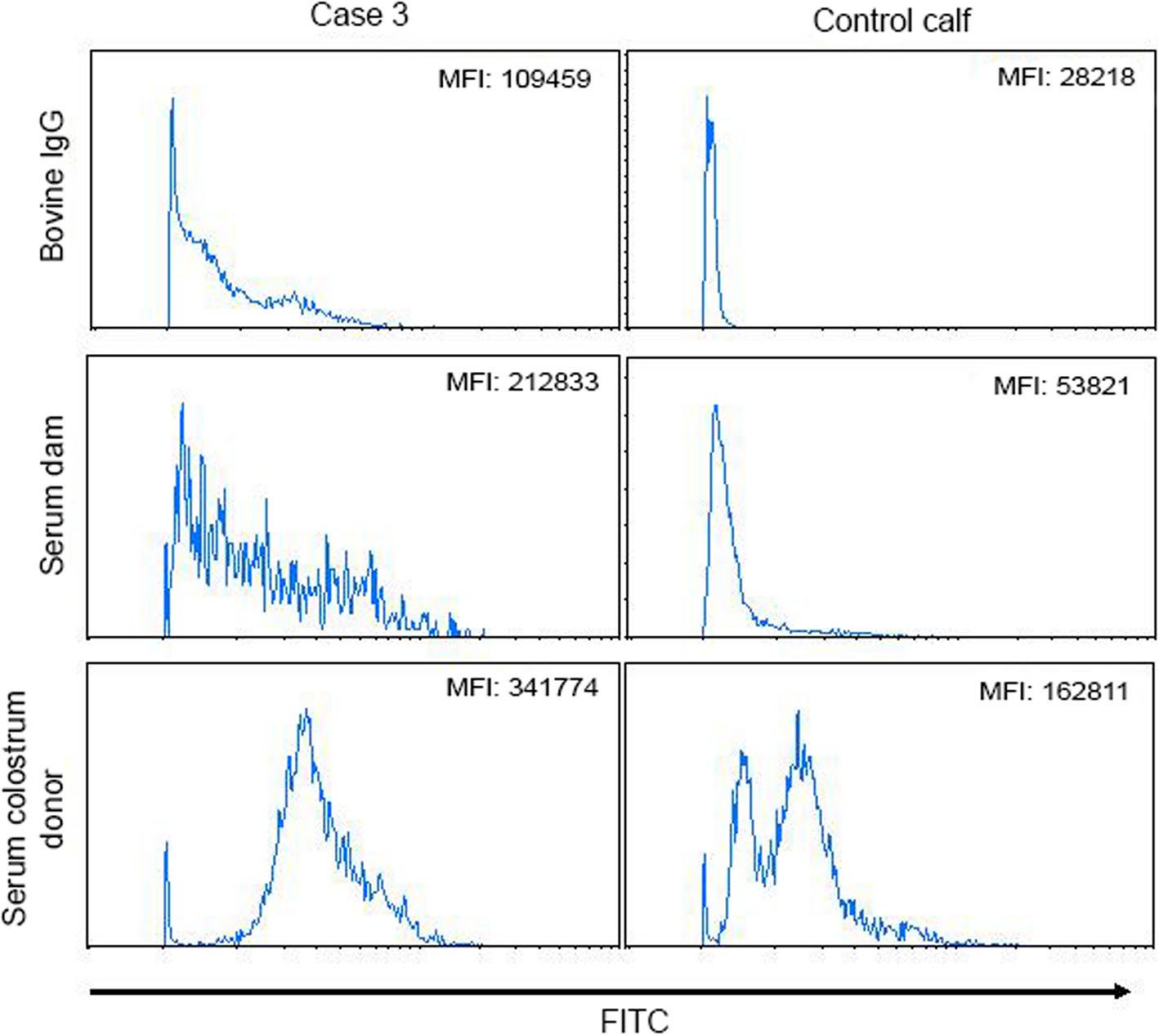
Leukocytes from two calves (case 3 and a healthy control calf) were stained with serum from the dam of case 3 or a colostrum donor of case 3. As a control the leukocytes were stained with bovine IgG. Binding of bovine IgG to the cells was detected with an FITC-conjugated anti-bovine IgG. The mean fluorescence intensities (MFI) of the leukocytes after incubation with the sera or bovine IgG are given in the histograms. MFI of PBS control staining: case 3: 101033; control calf: 27791

The level of antibody binding was measured by flow cytometry. After disinfection of the skin, blood was drawn from the jugular vein with a vacutainer system (Venoject®; Terumo, Leuven, Belgium) into a silica containing BD Vacutainer® SST II™ tube (BD, Erembodegem, Belgium). Calf leukocytes were isolated as previous described by Pardon et al. [19]. Upon isolation, cells were fixed in paraformaldehyde (4%) and stained with sera of the dams (cases 1–3) and serum from a colostrum donor (case 3). To this end, leukocytes (1.0x10^5^/mL) were transferred to wells of a conical bottomed 96-well microtiter plate. Cells were incubated 30 min on ice with two different serum samples diluted 1/50 in phosphate-buffered saline (PBS). As a control, leukocytes were stained with PBS only (control) or with bovine IgG at 0.2 mg/ml (case 3). Cells were then washed with PBS and stained with fluorescein isothiocyanate (FITC)-conjugated F(ab’)2 fragment of rabbit anti-bovine IgG (H&L) (1/500 dilution, Tebu-bio nv, Boechout, Belgium). Upon incubating 30 min on ice, protected from light, cells were washed with PBS and analysed using a FACSAria III flow cytometer (BD Biosciences, Erembodegem, Belgium) or a cytoflex (Beckman Coulter, Brea, CA, USA) (case 3). Doublets were excluded and cells were selected based on their size (FSC) and granularity (SSC). Data for at least 10.000 singlets were recorded using FACSDiva software (BD) or CytExpert (Beckman Coulter). Results from the flow cytometry, as shown in Figs. 1 and 2, revealed that the serum IgG from the dams binds to leukocytes isolated from their calves (case 1 and 2). Also for case 3 (Fig. 2), serum from the dam bound to leukocytes isolated from its calf, but not to leukocytes isolated from a healthy control calf. In addition, serum from the colostrum donor bound to the leukocytes isolated from case 3 as well as to the leukocytes isolated from the control calf.

### Therapy and case outcome

Case 1 died shortly after arrival. Case 2 was critically ill and received intravenous antibiotic treatment with 5 mg/kg/day enrofloxacin (Floxadil®, Animalcare Ltd., York, United Kingdom) and 80.000 IE/kg/d sodium benzylpenicilline (Penicilline 5.000.000 IE, Kela Pharma nv, Sint-Niklaas, Belgium). Also 0.6 mg/kg dexamethasonnatriumfosfaat (Rapidexon 2 mg/ml®, Dechra Regulatory BV, Bladel, The Netherlands) was injected intramuscularly. The day after arrival, the haematocrit of the calf decreased even further to 10% and a blood transfusion was given. One litre of blood from an adult Holstein-Friesian cow, housed at the clinic, was transfused to the calf using 5.5 g of sodium citrate anhydrate (Natrii Citras ®, Fagron N.V., Waregem, Belgium) as anti-coagulant. The clinical condition improved slightly and treatment with enrofloxacine and natriumbenzylpenicilline was continued. After two days hospitalisation the animal’s clinical condition deteriorated. Bronchopneumonia was diagnosed during the follow-up ultrasonographic examination. Antibiotic treatment was revised and switched to 15 mg/kg spectinomycine-lincomycine (Emdactilin 150®, Emdoka bv, Hoogstraten, Belgium) and the non-steroidal anti-inflammatory drug, 1.1 mg/kg flunixini megluminum (Emdofluxin 50®, Emdoka bv, Hoogstraten, Belgium) was given. The clinical condition further decreased and the calf died later that day. At admission, case 3 could still stand, but his general condition worsened soon after entry. A broad spectrum intravenous antibiotic treatment consisting of enrofloxacin and sodium benzylpenicilline was administered and a one litre blood transfusion was conducted. Very shortly after the blood transfusion, the calf deceased.

### Necropsy

**Fig. 3:**
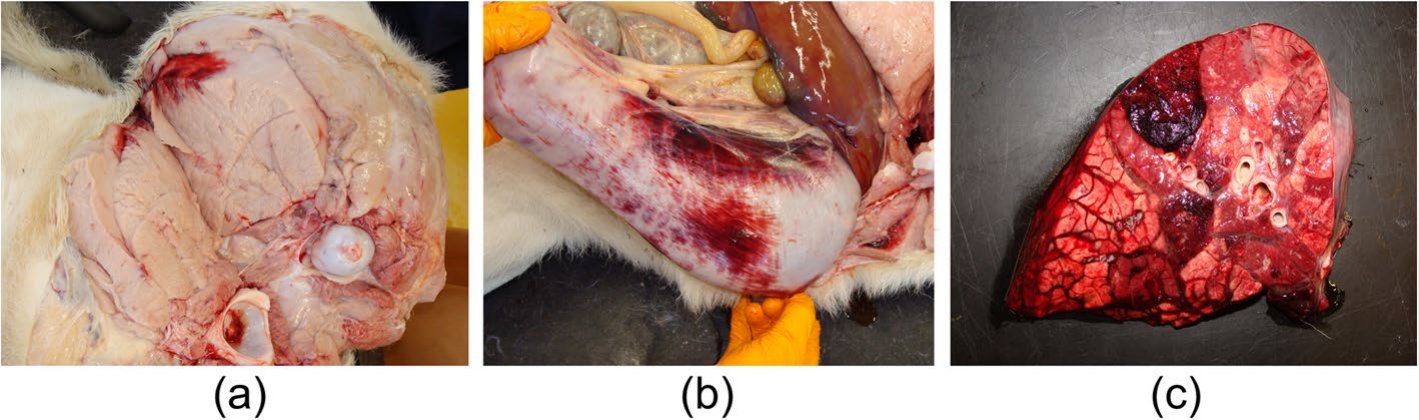
Pictures taken during necropsy of case 1 and 2. **a** Case 1. Severe pallor of the hind limb musculature due to anaemia, with a focal intramuscular ecchymosis. **b** Case 1. Extensive serosal haemorrhage on the abomasum. **c** Case 2. Lung at cut surface. Haemorrhagic bronchopneumonia characterized by lobular, pink discoloration with haemorrhage and firm aspect upon palpation

Necropsy was performed according to a standard protocol. All three calves were necropsied within 24 h after death. At gross examination, they had a pronounced anaemic appearance and displayed extensive generalized haemorrhages (petechiae and ecchymoses), shown in Fig. 3a. Haemorrhages in varying severity were present on the skin, mucosae, serosae (Fig. 3b) and muscles, but were most prominent at the subcutaneous peri-articular tissue and unilateral neck musculature in all three animals. The latter is most likely secondary to intramuscular injection(s). In case 2, a severe haemorrhagic bronchopneumonia and fibrinous pleuritis were present (Fig. 3b).

**Fig. 4:**
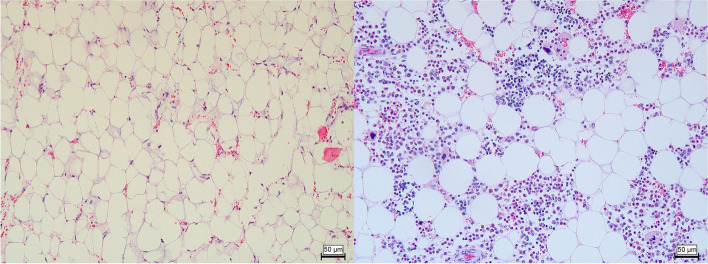
Bone marrow of case 1 (left) and a control (right). There is complete depletion of haematopoietic cells in case 1. Hematoxylin and eosin stain, 20x

**Fig. 5:**
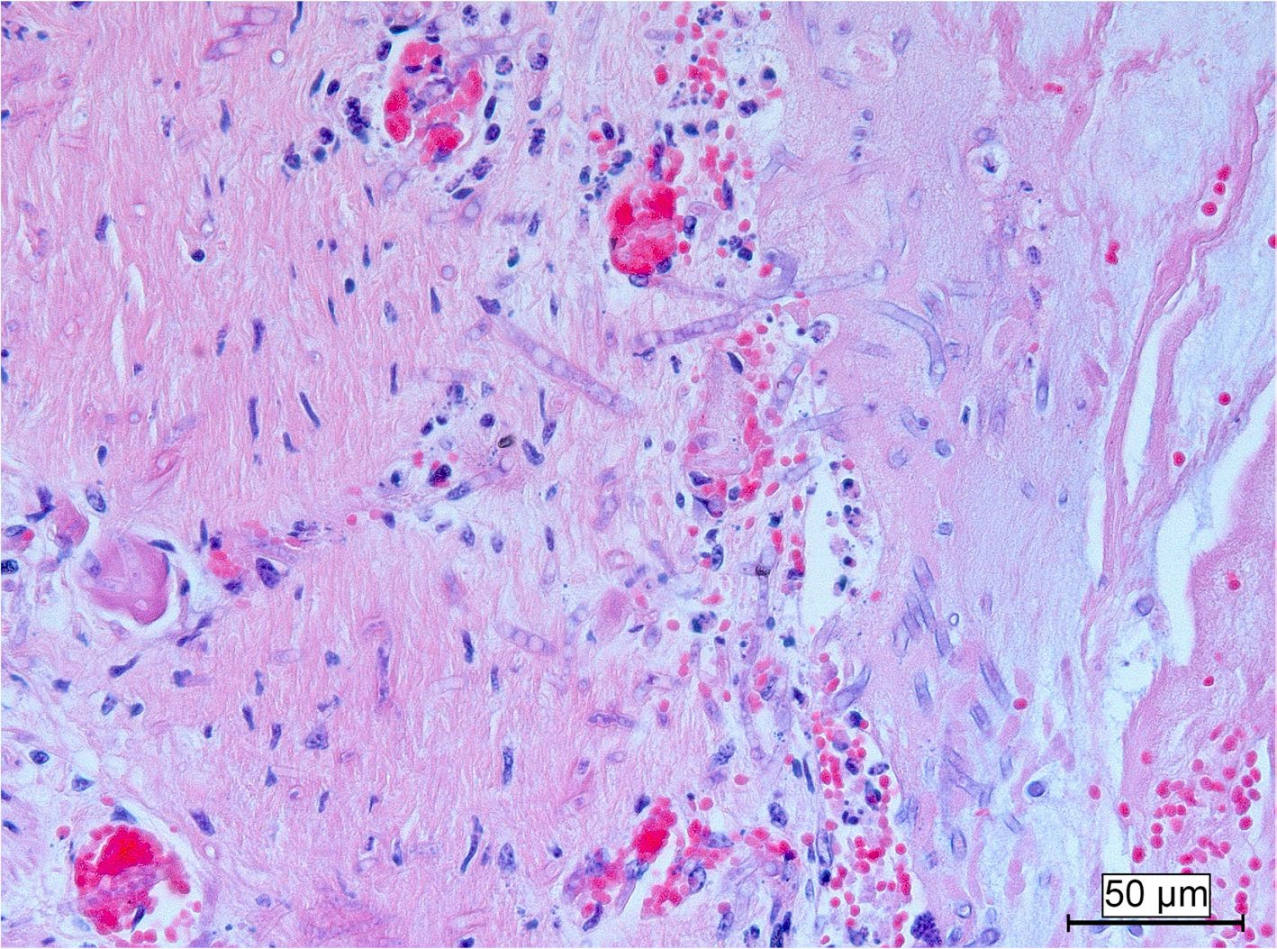
Lung of case 2. Extensive invasive growth of fungal hyphae in the bronchiolar wall with only few inflammatory cells. Hematoxylin and eosin stain, 20x

Histopathological examination of the lung, spleen, bone marrow, kidney and liver were performed for all three calves; the iliac lymph node was only sampled in case 1 and 3. Samples were fixed in 10% neutral buffered formaldehyde and routinely processed. Histologically, the bone marrow was characterized by a total absence of hematopoietic tissue in case 1 (Fig. 4) and severe loss in case 2 and 3. All cell populations were affected (total depletion). The spleen and lymph node displayed moderate (case 2 and 3) to severe (case 1) depletion of lymphoid cells of the periarteriolar lymphoid sheaths and lymph node cortex. Lung tissue of case 2 confirmed the gross diagnosis, the histological appearance was characterized by necrosuppurative bronchiolitis with intralesional growth of fungal hyphae in the bronchiolar and peribronchiolar tissue (Fig. 5). Also noticeable intra-alveolar and interlobular haemorrhages and oedema were present with low numbers of neutrophils and macrophages.

## Discussion and conclusions

This report describes three cases of pancytopenia in suckler calves, clinically indistinguishable from BNP. In the diagnostic work-up, known aetiologies were excluded. Thrombocytopenia is far more frequent as a cause of bleeding disorders in calves than pancytopenia, which fairly limited the differential list.

One of the most important differential diagnosis is BVDv. At birth, BVDv was tested by antigen-ELISA on ear notch as requested by the Belgian BVDv eradication program. This test reaches a sensitivity of 100% (95% CI: 90.50–100) [34]. The fact that the calf was not tested for BVDv at the peak of clinical signs, could be regarded as a limitation of this study. However, given that the farm of case 1 and 2 has been documented BVDv free since 2010 with regular monitoring, i.e. ear notch antigen ELISA of each newborn calf, makes involvement of BVDv very unlikely.

Bacteremia was evidenced in all calves. The presence of bacteria in the bloodstream can induce disseminated intravascular coagulation (DIC) [35]. An extensive DIC may cause thrombocytopenia and explain the increased bleeding tendency. As DIC involves systemic activation of the coagulation system and plasma levels of D-dimer increase by activation of the fibrinolytic system, the determination of D-dimers could have provided added value in case 1 and 2 and can be considered as a limitation in this report. In case 3, no increased values of D-dimers were observed. Although elevated D-dimer levels are not specific for DIC, levels are generally much higher than with other etiologies of clot formation [36, 37]. Also, primary pathogens such as *Pasteurella multocida,* renowned for haemorrhagic septicemia, could not be identified with blood culture [38, 39]. Given the extreme leukopenia, bacteremia is likely secondary, as previously seen in BNP calves [3]. The opportunistic nature of the isolated bacteria substantiates this. Nevertheless, the initial treatment of the ambulatory veterinarian in case 1 and 2, which could have eliminated a primary pathogen, cannot be neglected. In addition, the MDR bacteria isolated in these cases were resistant against penicillin with disk diffusion testing. Disk diffusing for gentamicin was not done. The authors assume this was done since both bacteria were gram positive and natural resistance was suspected, yet aminoglycosides are known to have activity against certain Staphylococcus species [40, 41]. Next to this, the medium in the BD Bactec™ Peds Plus™ and Plus Aerobic medium™ contains resins for antibiotic neutralization, which can give secondary pathogens, as isolated here, the opportunity to proliferate. We did not test for fungi by blood culture in case 1 and 2, but given that there was fungal pneumonia in one case, potentially also fungal sepsis occurred in these animals. Other known etiologies of bleeding disorders, like rodenticide poisoning [42] or hereditary diseases [43–45] are more associated with thrombocytopenia rather than pancytopenia. Also intoxication due to certain medication can be excluded in these cases simply due the fact that no medication was given before the onset of the clinical signs. There was also no mention of medication used during pregnancy except the vaccination.

By using flow cytometric assays, we evidenced binding of the alloimmune antibodies from the dam on leukocytes from the calves. Given the bone marrow depletion, it is likely that these antibodies also bind to precursors of the different cell lineages in the bone marrow, as was observed with BNP. In the past, the use of Pregsure® BVD was associated with BNP. In the last 10 years, this vaccine had not been used in the affected farm. An extensive enquiry exploring the use of colostrum or antibody supplements was performed in the last years, but none had been used on these farms. Mortality was high in the three described cases in this report, whereas the BNP cases occurring in the tail of the epidemic after removal of the vaccine had high survival chances [3, 46]. This raises the suspicion that the animals in this case report were again confronted with a higher dose of alloantibodies.

Neonatal isoerythrolysis, an immune-mediated haemolytic anaemia, develops in neonatal animals following ingestion of colostrum containing antibodies against antigens on their erythrocytes. Hemolytic disease is described in cattle, however these cases were induced by vaccines against anaplasmosis and babesiosis which contained whole blood or erythrocyte membrane fragments [47]. To the author’s knowledge, no cases have been published whereby hemolytic disease was induced by maternal synthetization with foetal red blood cells during previous parturitions, which is commonly described in horses [48]. Leakage of fetal erythrocytes during pregnancy is unlikely in cattle due to the cotyledonary epitheliochorial placenta which represents the least intimate association between maternal and fetal tissues. Also blood transfusions can induce maternal antibody production [49], but neither the mothers nor the colostrum donating dams received a blood transfusion during their lifetime which excludes a mismatched blood transfusion as an initiator. Next to this, no clinical signs of hemolysis such as icterus, hemoglobinemia or hemoglobinuria were seen in these cases. The observed anaemia can be explained by ongoing blood loss at the level of the many hemorrhages due to clothing failure.

Although no firm conclusions can be drawn from a single case report, vaccination as source of alloantibodies is an important hypothesis to explore. Both the Pregsure® BVD as the bluetongue vaccines use an adjuvant containing very potent saponins, in particular Quil A, of which some researchers believed to have contributed to the severity of BNP as it induced high and persistent alloantibody titers [17, 22]. In both herds, a lot of different vaccines were used. If we compare the vaccination strategies of both farms there are two vaccines that were used in both farms, namely a bluetongue vaccine and a vaccine against bRSV and PI-3. The two bluetongue vaccines, Bluevac BTV® and Bovilis Blue-8®, are the same vaccine and only differ from each other by name. However, it is not possible to draw any conclusions out of this preliminary information. As with BNP, a genetic component may also be involved in the pathology of the calves [18]. Involvement of the bull in these cases was not assessed in depth as the calves did not have a common sire and a further genetic determination was not within the scope of this case report. However, Belgian Blue is an intensively selected cattle population and as a consequence of the selection process in this breed the level of inbreeding is relatively high [50].

In conclusion, this report describes three cases of alloimmune mediated pancytopenia in suckler calves, resembling previous BNP cases. The immunoassay clearly demonstrates that the clinical conditions in these cases are immune-mediated through antibodies received from the dams via colostrum. Although an exact aetiology cannot be evidenced in a case report, taking the above reasoning into account, vaccine induced antibodies are among the more likely causes. It is worrisome that the case fatality rate was 100% in these three cases, which is reminiscent to the 2007–2010 outbreak of BNP. This report shows that increased surveillance and pharmacovigilance towards bleeding syndromes in calves is needed again.

## Data Availability

The datasets used and/or analysed during the current study are available from the corresponding author on reasonable request.

## Abbreviations

BVDv: Bovine viral diarrhoea virus
BNP: Bovine neonatal pancytopenia
BRSV: Bovine respiratory syncytial virus
PI-3: Para-influenza 3
MDR: Multi drug resistant
ELISA: Enzyme-linked immunosorbent assays
MALDI-TOF MS: Matrix assisted laser desorption/ionisation time-of-flight mass spectrometry
PBS: Phosphate-buffered saline
FITC: Fluorescein isothiocyanate
MFI: Mean fluorescence intensity
DIC: Disseminated intravascular coagulation
